# Resumé of Some Experiments to Determine the Quantity of Blood in the Body

**Published:** 1849-08

**Authors:** James Blake

**Affiliations:** Professor of Anatomy in St. Louis University


*Resumé of some Experiments to determine the quantity of blood in the body.* By JAMES BLAKE, M. D., F. R. C. S. E., *Professor of Anatomy in St. Louis University.* (Communicated in a letter to Prof. Dunglison.)

The experiments were performed by injecting a weighed quantity of sulphate of alumina into the veins, and analyzing a weighed portion of the blood. As the salt had time to be well mixed with the blood before the animal died, such an analysis would enable us to determine the whole quantity of blood with which the salt had been mixed. The only error which might arise would be from a portion of the salt having combined with some of the tissues, or having been rapidly excreted, and thiswould only affect the result in one direction, viz. in furnishing a greater quantity of blood than really exists.

The results of my experiments would lead to the conclusion that no such source of error exists, as I find by this method that the weight of blood in the body of a dog does not amount to more than between one-eighth or one-ninth part of the weight of the animal, an estimate much lower than that which is generally received as the quantity of blood in an animal. That this, however, is nearer the truth, is probable, from the consideration of the velocity of the circulation and the capacity of the heart, as on the generally received opinion on the quantity of the blood, it is difficult to imagine how it can circulate so rapidly.

